# Synthetic Potato Systemin Enhances *Solanum tuberosum* Resistance to Potato Virus Y

**DOI:** 10.3390/biom16091353

**Published:** 2026-09-17

**Authors:** Alexander Skripnikov, Tatiana Suprunova, Michael Taliansky, Natalia O. Kalinina

**Affiliations:** 1 Shemyakin–Ovchinnikov Institute of Bioorganic Chemistry, Russian Academy of Sciences, Miklukho-Maklaya Street 16/10, Moscow 119997, Russia; 2 Biology Department, Lomonosov Moscow State University, Leninskiye Gory 1, Building 12, Moscow 119234, Russia; 3 Doka-Gene Technologies Ltd., Moscow Region, Rogachevo 141880, Russia; 4 Belozersky Institute of Physico-Chemical Biology, Lomonosov Moscow State University, Leninskiye Gory 1, Building 40, Moscow 119234, Russia

**Keywords:** plant peptides, systemin, foliar application, crop protection, potato, potato virus Y

## Abstract

In recent years, short plant peptides have emerged as highly promising, innovative bioregulators of crop growth and resilience. Activating systemic defense using synthetic plant peptides represents a potent strategy to improve crop resistance against pathogenic threats. Potato virus Y (PVY) causes devastating economic losses in potato (*Solanum tuberosum*) crops. In this study, we investigated the protective activity of synthetic analogs of systemin, a classic defense peptide originally discovered within the Solanaceae family. We synthesized the putative potato octadecapeptide systemin (AVHSTPPSKRDPPKMQTD) along with two 17-mer variants, each truncated by a single amino acid residue at either the N- or C-terminus, for foliar application. The defensive efficacy of these peptides was evaluated using inoculation and monitoring viral accumulation under controlled growth chamber conditions. Foliar application of all three synthetic peptides significantly reduced the accumulation of the PVY^O^ ordinary strain over a one-month post-infection period with comparable efficacy. Furthermore, we demonstrated the antiviral effect of the synthetic octadecapeptide against the exceptionally devastating and widespread recombinant PVY^NTN^ strain. These findings suggest that synthetic derivatives of potato systemin could serve as potential tools for developing eco-friendly antiviral strategies for sustainable crop protection.

## 1. Introduction

Short peptides play essential roles in regulating plant growth, development, and responses to environmental stress, including pathogen attacks [1,2,3]. Synthetic analogs of identified and putative elicitor peptides are emerging as promising biostimulants that enhance crop protection and productivity in an eco-friendly manner, offering a sustainable alternative to conventional agrochemicals [4]. A primary advantage of these short peptides is their application as aqueous foliar sprays—the most practical and widely used method of plant treatment—at ultra-low concentrations, which makes them exceptionally eco-friendly. The choice of femtomolar concentration range was directly guided by pioneering studies on tomato systemin, where protective biological activity was demonstrated at femtomolar thresholds [5,6], as well as recent studies conducted within a similar range [7].

Among functional peptides well-established by mass-spectrometry is tomato systemin—the first peptide isolated from plants. It is an 18-amino-acid peptide AVQSKPPSKRDPPKMQTD cleaved from its 200 a.a. precursor protein, prosystemin [5]. Systemin functions upstream the jasmonate pathway [5,8,9], acting locally at the site of stress to amplify jasmonate biosynthesis to the threshold levels required for systemic defense responses [10,11,12]. Systemin perception depends on a pair of distinct cell-surface receptor kinases, SYR1 and SYR2, which belong to a large family of plant receptor kinases with extracellular leucine-rich repeat (LRR) domains [13,14,15,16]. Within this system, SYR1 acts as a genuine systemin receptor that binds the immunogenic signal with high affinity and specificity [17]. Recently, four additional systemin paralogs encoded within a single gene cluster have been identified in tomato plants, three of which function as newly discovered agonistic systemins, while the fourth—termed antiSYS—acts as a potent natural antagonist of the systemin receptor [18].

While tomato systemin has been confirmed by mass spectrometry [5,19], the precise amino acid sequence of mature potato systemin has yet to be verified by MS analysis—the only definitive method for regulatory peptide identification [20]. Based on sequence alignment, several 18-amino-acid peptide variants (octadecapeptides) were proposed as putative systemins across Solanaceae species, including black nightshade *Solanum nigrum* (AVRSTPPPKRDPPKMQTD) and pepper *Capsicum annuum* (AVHSTPPSKRPPPKMQTD), with the potato *Solanum tuberosum* systemin I and II (StSys I and StSys II) sequences designated as AVHSTPPSKRDPPKMQTD and AAHSTPPSKRDPPKMQTD, respectively [12,21,22]. For clarity and consistency, all subsequent references to potato systemin, StSys I, or its variants in this study imply their putative status or synthetically reproduced sequences. In potato plants, however, it is highly plausible that natural precursor processing does not strictly mandate a uniform 18-amino-acid peptide product. Because the ultimate chain length of a regulatory peptide is governed by in vivo proteolytic boundaries, predicting cleavage sites or identifying the responsible proteases remains a major challenge in plant biology [20]. Given that the exact proteolytic processing of potato systemin lacks definitive mass-spectrometric confirmation, it is biochemically conceivable that its functional length deviates from the putative 18-mer paradigm.

Structural variability of short regulatory peptides is functionally significant; early structure–activity relationship studies on tomato systemin demonstrated that the omission of even a single N-terminal alanine reduced its biological activity 300-fold [23]. Given this potential dependency of bioactivity on peptide sequence and structure, incorporating truncated variants alongside the putative, bioinformatically postulated peptide provides a robust framework for a focused screening approach. This strategy paves the way for a preliminary evaluation of how minor terminal cleavage variations affect the peptide’s protective efficacy. Targeted truncation of terminal amino acids represents a rationally justified approach for the initial stage of peptide bioactivity investigations, as it circumvents the labor-intensive demands of high-throughput screening while remaining far more informative than testing of a single putative peptide.

Among all economically important potato viruses, this study focuses on potato virus Y (PVY) as the most hazardous and widespread pathogen, which continues to pose a major challenge to global agriculture due to the rapid emergence and spread of recombinant strains [24]. To explore the crop-protective activity and functional boundaries of potato systemin derivatives, this study utilized experimental approaches based on the inoculation of plants and the evaluation of viral accumulation dynamics under controlled growth chamber conditions. Plants were treated via foliar spraying with an aqueous peptide solution. First, we conducted these experiments to evaluate the biological activity of the putative octadecapeptide StSys I alongside two truncated 17-mer variants (missing either the N-terminal Ala or the C-terminal Asp) against the PVY^O^ strain, monitoring viral accumulation dynamics following inoculation. Second, we expanded our studies to investigate whether the protective capacity of the octadecapeptide StSys I could suppress the more aggressive and widespread PVY^NTN^ strain. Overall, this preliminary study demonstrates that synthetic potato systemin derivatives possess protective potential and can be further developed as a signaling biostimulant to enhance potato resistance against viral pathogens.

## 2. Materials and Methods

### 2.1. Plants, Virus and Growth Conditions

In vitro-propagated *Solanum tuberosum* L. cv. Indigo plants (breeding of Doka-Gene Technologies Ltd., Rogachevo, Russia) were used to evaluate the effect of the synthetic peptide systemin derivatives on potato resistance to potato virus Y.

Potato plants were grown in soil from in vitro-propagated virus-free plantlets in a controlled environment chamber (Pol-Eko-Aparatura, Wodzisław Śląski, Poland) which was set at a photoperiod of 16/8 h day/night with a relative humidity of 40% and a light fluence of 250 µmol m^−2^ s^−1^. Two-weeks post-transplanting, Indigo plants were used for experiments.

Ordinary PVY strain O (PVY^O^) [25] was maintained and propagated in *Nicotiana tabacum* [26]. The necrotic recombinant strain PVY^NTN^ was originally isolated from potato fields in the Moscow Region [27], identified via sequencing by Doka-Gene Technologies Ltd. (Rogachevo, Russia), and kindly provided for this study. Both viral strains were propagated and maintained in *Nicotiana tabacum* plants under the same controlled conditions as the potato plants. For the experiment, three fully expanded leaves per plant were inoculated with PVY (crude sap from infected plants) by gently rubbing the infectious sap from *Nicotiana tabacum* onto carborundum-dusted target tissues using a standard sap inoculation protocol.

### 2.2. Peptide Synthesis and Treatment

The octadecapeptide StSys I (AVHSTPPSKRDPPKMQTD) and its two 17-mer truncated variants, StSys I-ΔD (AVHSTPPSKRDPPKMQT, lacking the C-terminal Asp) and StSys I-ΔA (VHSTPPSKRDPPKMQTD, lacking the N-terminal Ala), were synthesized via solid-phase peptide synthesis (SPPS) using the standard N-α-Fmoc strategy. The crude peptides were purified by preparative high-performance liquid chromatography (HPLC). Peptide purity (≥95%) and identity were verified by analytical reversed-phase HPLC (RP-HPLC) and matrix-assisted laser desorption/ionization time-of-flight mass spectrometry (MALDI-TOF/TOF-MS), with ultimate sequence confirmation provided by Orbitrap nano-liquid chromatography tandem mass spectrometry (nano-LC-MS/MS).

The synthetic peptides were dissolved in sterile distilled water to prepare a 10 fM working solution [5,6]. Foliar application of the synthetic peptides was performed using a handheld fine-mist nebulizer. Each leaf of the plant received 3–4 sprays (approximately 200 μL per leaf), resulting in a total volume of roughly 1 mL per plant to ensure uniform coverage of all aerial parts. This treatment protocol was applied across both experimental series targeting the PVY^O^ and PVY^NTN^ strains. Control plants were sprayed with an equivalent volume of sterile distilled water.

### 2.3. Experimental Design

The experimental setup consisted of two distinct testing schemes designed to evaluate the curative, protective, and combined efficacy of the synthetic peptides under controlled conditions. Experiments were independently repeated three times. Each treatment group included thirteen biological replicates (*n* = 13).

First Experimental Series (Supplementary Figures S1 and S2). This series evaluated the curative activity of the octadecapeptide StSys I and its two 17-mer truncated variants, StSys I-ΔD and StSys I-ΔA, against the PVY^O^ strain. Plants were inoculated first, and the foliar peptide treatment was administered strictly 24 h post-inoculation.

Second Experimental Series (Supplementary Figure S3). This series evaluated the sequential protective and curative activity of the 18-mer peptide StSys I against the PVY^NTN^ strain. The experimental groups were subjected to a weekly spraying regimen administered as follows:

- Treatment 1 (Single Preventive): Received a single preventive foliar spray 24 h prior to inoculation.
- Treatment 2 (Preventive + Single Curative): Received the initial preventive spray plus one curative foliar spray administered 24 h post-inoculation.
- Treatment 3 (Preventive + Two Curative): Received the full Treatment 2 sequence plus a second post-infection curative spray 1 week post-inoculation.
- Treatment 4 (Preventive + Three Curative): Received the full Treatment 3 sequence plus a third post-infection curative spray 2 weeks post-inoculation.

### 2.4. Viral Accumulation Assessment

ELISA. To monitor viral accumulation, leaf samples were taken from systemic (non-infected leaves) at the designated post-infection time points at 14, 21, and 28 days post-inoculation (DPI) in the first experimental series, and at 21, 28, and 35 DPI in the second series and analyzed by ELISA using the BIOREBA ELISA Complete Kit (BIOREBA AG, Reinach, Switzerland) according to standard diagnostic protocols. Raw optical density values were converted to calibrated viral accumulation units using a cubic regression model.

RNA Extraction and Real-Time Quantitative PCR (RT-qPCR)

Total RNA was extracted from frozen potato systemic leaf tissues at 7, 14 and 21 DPI in the first experimental series (Supplementary Figure S2) using TRI REAGENT (Sigma-Aldrich, St. Louis, MO, USA) and suspended in DEPC-treated water. DNase-treated RNA was reverse-transcribed into cDNA using the SuperScript™ First-Strand Synthesis System for RT-PCR (Invitrogen, Carlsbad, CA, USA). SYBR Green-based RT-qPCR analysis of PVY RNA was performed using the virus-specific primers PVY^O^-F (5′-TATGATGGATTTGGCGACCACTTGT-3′) and PVY^O^-R (5′-TAAACTAGGCAGCTCTGCATCATG-3′) [26] at a final concentration of 400 nM (E = 95.7%). Primer pairs for the internal reference genes, StEF-1α and StCOX, were verified/designed using both Plant Genomics Resource Phytozome and PRIMER EXPRESS software v3.0 (ThermoFisher Scientific, Waltham, MA, USA). The C_t_ values for PVY RNA were normalized against the average of the two reference genes.

### 2.5. Statistical Analysis and Graphical Representation

Statistical analyses and graphical representations were performed in Python 3.9.6 using the NumPy v2.0.2, Matplotlib v3.9.4, Statsmodels v0.14.6, SciPy v1.13.1 and Pingouin v0.5.5 libraries. Mean values were analyzed via two-way mixed-effects repeated measures ANOVA followed by Tukey’s Honestly Significant Difference (HSD) post-hoc test to identify significant differences between peptide-treated groups and the control. Differences were considered statistically significant at *p* < 0.05.

## 3. Results

### 3.1. Curative Antiviral Efficacy of Synthetic Systemin Variants Under Controlled Conditions

**Figure 1:**
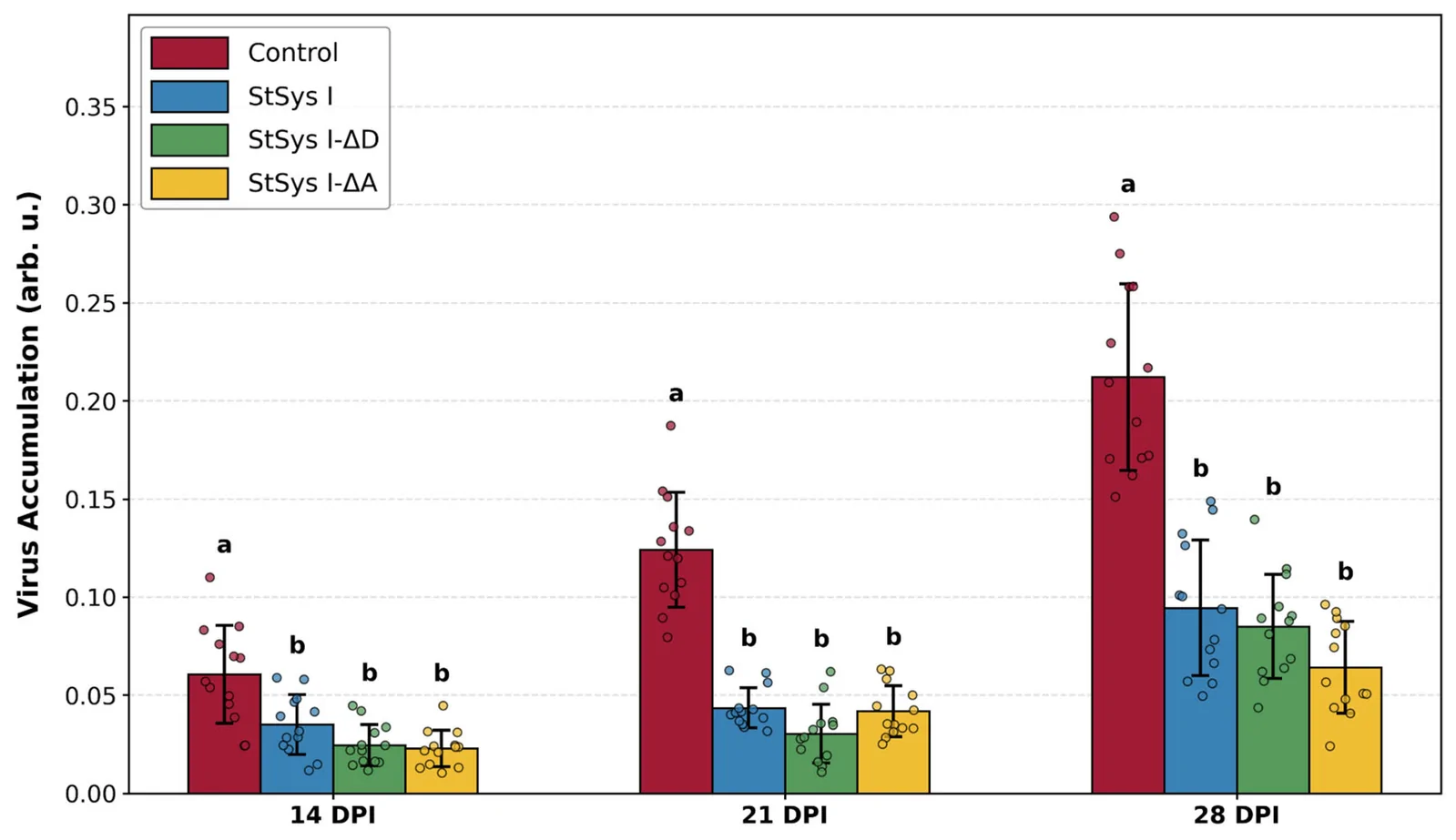
Accumulation dynamics of Potato virus Y (PVY^O^ strain) in systemically infected leaves of potato plants (*Solanum tuberosum* cv. Indigo) at 14, 21, and 28 (DPI). Plants were treated curatively 24 h post-infection with the synthetic 18-mer potato systemin (StSys I, 10 fM), its truncated 17-mer variants (StSys I-ΔA and StSys I-ΔD, 10 fM), or water (Control). Data represent mean ± SD from *n* = 13 independent biological replicates. Different letters indicate statistically significant differences (*p* < 0.05, Tukey HSD).

To evaluate the immediate post-infection protective potential of the synthetic peptides, viral accumulation dynamics were monitored in the foliage of *S. tuberosum* cv. Indigo at 14, 21, and 28 DPI using the ELISA method. Foliar treatment with all three systemin variants 24 h post-inoculation led to a decrease in PVY^O^ accumulation compared to the control (Figure 1). At 14 DPI, the full-length 18-mer peptide StSys I substantially lowered the viral load. The N-terminally truncated variant (StSys I-ΔA) and the C-terminally truncated variant (StSys I-ΔD) demonstrated comparable curative efficacy during this stage, resulting in a reduction of viral accumulation. As the infection in the control group progressed to 21 DPI, the inhibitory effect across all three peptide-treated groups remained significant. The viral accumulation in all peptide-treated groups exhibited a substantial reduction compared to the control group. By the end of the observation period at 28 DPI, the effect of the 17-mer truncated variants was similar to that of the full-length octadecapeptide, demonstrating comparable viral suppression. These data suggest that the truncation of a single terminal amino acid residue does not diminish the protective capacity of the peptide at ultra-low concentrations, confirming that both the 18-mer and 17-mer derivatives maintain their ability to trigger a reduction in PVY^O^ accumulation (Figure 1). The ELISA results from the first experimental series were independently validated using RT-qPCR analysis of PVY^O^ RNA accumulation at 7, 14, and 21 DPI (Supplementary Figures S2 and S4). The molecular data supported the trends observed via ELISA, showing that all peptide treatments successfully reduced viral RNA accumulation compared to the infected control group. Due to the high sensitivity of RT-qPCR, the differences between the control and treated plants appeared to be more pronounced at the transcription level. Based on this validation, the ELISA method was continued to monitor the second large-scale experimental series against the PVY^NTN^ strain.

### 3.2. Evaluation of Synthetic Systemin Protective and Curative Efficacy Against the Necrotic PVY^NTN^ Strain

**Figure 2:**
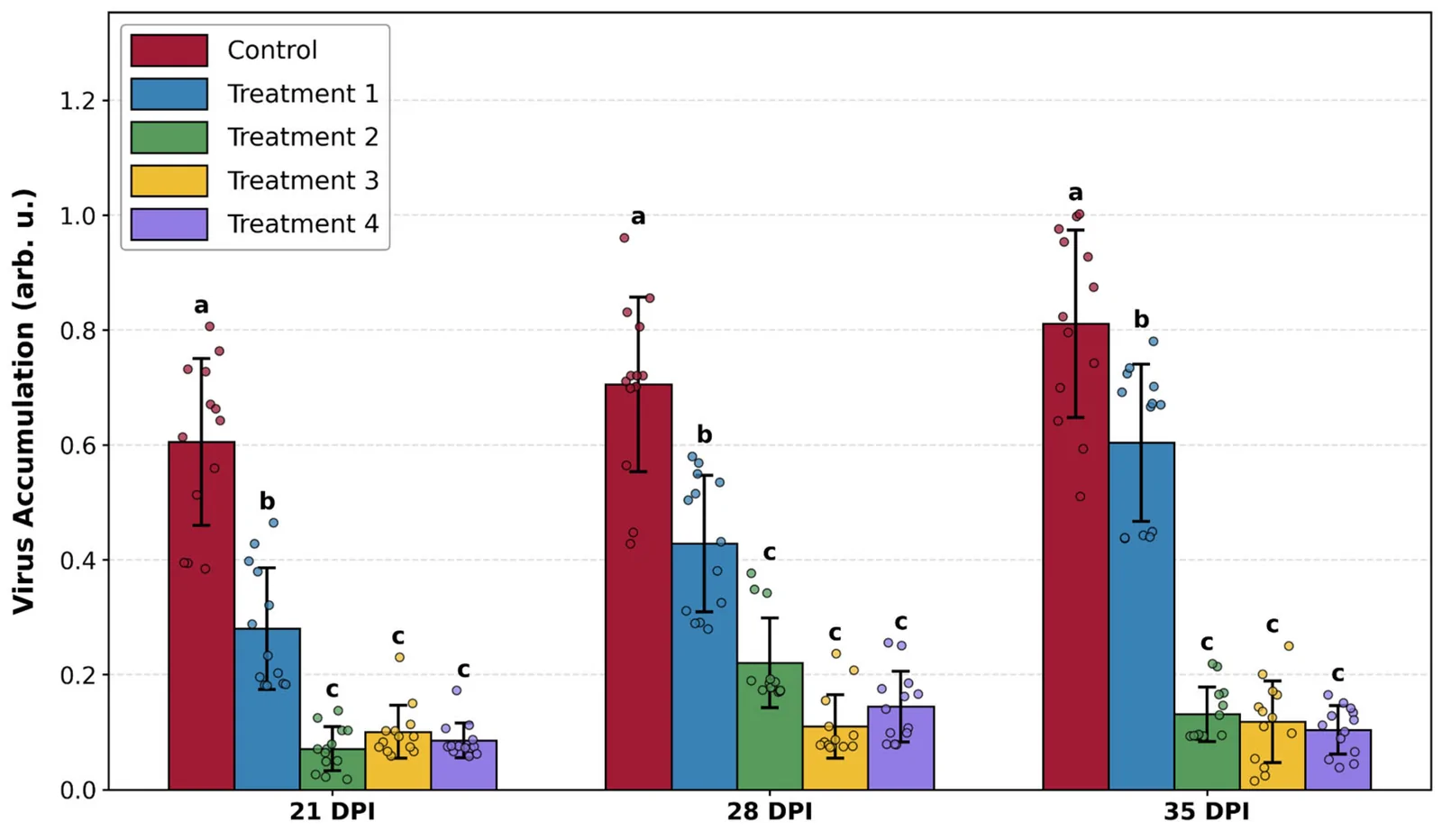
Accumulation dynamics of Potato virus Y (PVY^NTN^ strain) in systemically infected leaves of potato plants (*Solanum tuberosum* cv. Indigo) at 21, 28, and 35 DPI. Plants were subjected to consecutive, sequential foliar spray treatments using the synthetic 18-mer potato systemin (StSys I, 10 fM) or water (Control). Treatment timelines were administered as follows: Treatment 1 received a single preventive spray 24 h prior to inoculation; Treatment 2 received the preventive spray plus an initial curative spray 24 h post-inoculation; Treatment 3 received the previous sprays plus a second curative spray one week post-inoculation; and Treatment 4 received the full sequence plus a third curative spray two weeks post-inoculation. Data represent mean ± SD from *n* = 13 independent biological replicates. Different letters indicate statistically significant differences (*p* < 0.05, Tukey HSD).

In the second experimental series, an expanded evaluation was performed using the recombinant PVY^NTN^ strain. This chronological timeline was designed to simulate standard curative treatments in potato cultivation, incorporating multiple consecutive peptide applications over a 35-day cycle. Based on the findings from the first experimental series against PVY^O^, all three peptide variants had demonstrated comparable protective efficacy. Therefore, to minimize experimental complexity during this large-scale sequential testing, the parental 18-mer StSys I was selected as the sole representative for the evaluation. For this purpose, viral accumulation was monitored at 21, 28, and 35 DPI across plants subjected to consecutive foliar peptide sprays (see Figure 2 and Figure S3). The data revealed a significant reduction in viral accumulation following the octadecapeptide StSys I foliar spraying among the experimental groups with curative Treatments 2–4. Post-hoc comparisons demonstrated that a single preventive foliar spray alone (Treatment 1) resulted in a reduction in viral accumulation at 21 DPI compared to the untreated infected control plants. However, without subsequent curative treatments after this single preventive peptide spraying the viral load in the Treatment 1 group showed continuous growth at 28 and 35 DPI, while remaining statistically distinguishable from the viral accumulation observed in the control group. In contrast to the first group (Treatment 1), continuing the treatments after the initial priming with successive post-infection curative foliar sprays yielded stable long-term protection. Plants that advanced further through the sequential timeline, receiving multiple consecutive sprays (Treatments 2, 3, and 4), showed a reduction in viral accumulation throughout the entire 35-day cycle. At 21, 28 and 35 DPI, the groups that had received the curative sequential spray applications exhibited a significant reduction in viral accumulation compared to the control group. These results suggest that while a single preventive foliar spray provides only an attenuated effect, sequential curative peptide applications maintain protection of potato plants against the highly aggressive recombinant PVY^NTN^ strain under controlled conditions (Figure 2).

## 4. Discussion

In recent years, short plant elicitor peptides have emerged as highly efficient bioregulators in plant biology research and a focal point for innovations in crop production. Putative 23-amino-acid analogs of Pep elicitor peptides, originally discovered in Arabidopsis, have been bioinformatically identified in potato. Currently, these molecules are being investigated as potential protective agents to suppress potato nematodes [28] and fungal pathogens [29,30]. Systemins were discovered decades ago as the pioneering examples of plant peptide hormones; nevertheless, they are currently experiencing significant interest [12,18,31]. Postharvest application of synthetic tomato systemin increases fruit resistance to *Botrytis cinerea* via stress-related MAPK pathways [32]. At picomolar and femtomolar concentrations, systemin application to tomato plants not only enhances their resistance to fungal pathogens [7] but also increases salt tolerance [33]. The multifaceted activity of systemin complicates the elucidation of its molecular mechanisms, especially at ultra-low concentrations. Nevertheless, research into its elicitor effects continues to expand, even in the absence of a fully characterized signaling cascade. In this study, we undertook the first effort to elucidate their elicitor potential against plant viruses using model experiments with inoculation of Potato virus Y (PVY), one of the most devastating potato pathogens. Under controlled conditions, a single curative foliar spray administered 24 h post-infection was sufficient to trigger a prolonged defense response against the PVY^O^ strain, maintaining a substantial reduction in viral accumulation through 28 DPI (Figure 1). For PVY load assessment, we used ELISA, an analytical standard in potato virology widely adopted for large-scale sample analysis. To cross-verify ELISA assessment under our experimental conditions, PVY accumulation was measured via RT-qPCR analysis of viral RNA levels (Supplementary Figures S2 and S4). The transcript-level approach confirmed the suitability of ELISA for evaluating extensive sample series of PVY dynamics following peptide treatments. In the second experimental series, all treated potato plants received an initial preventive foliar spray 24 h prior to PVY^NTN^ inoculation. Over time, a single preventive spray alone (Treatment 1) proved insufficient for long-term protection, as viral accumulation increased by 28 and 35 DPI, although it remained lower than in the control group. The addition of a subsequent curative treatment 24 h post-inoculation (Treatment 2) provided stable antiviral activity, reducing the viral load at 21 DPI and maintaining it at low levels across the entire 35-day cycle. Additional peptide applications performed one and two weeks post-inoculation (Treatments 3 and 4) did not further enhance the effect observed with the single post-inoculation treatment (Treatment 2). Under these cumulative regimens, the viral load remained at a similarly reduced level at 21, 28, and 35 DPI, whereas viral accumulation in the control group progressively increased (Figure 2 and Figure S3). While the full sequence of four treatments yields substantial suppression of the necrotic strain, our data suggest that a two-spray regimen provided suppression of PVY^NTN^ over five weeks in this series of experiments. To optimize the timeline for antiviral potato peptide treatments, further investigations are required. Upon perception by its cognate receptor SYR1, systemin activates downstream signaling cascades tightly linked to jasmonic acid, a key regulator of plant defense responses under both biotic and abiotic stress [17,18]. In the context of our study, the synthetic systemin variants may trigger antiviral defense responses through these conserved signaling pathways. Future studies monitoring marker genes for jasmonic acid, ethylene, and salicylic acid are necessary to elucidate the underlying molecular mechanisms. While the efficacy of synthetic potato systemin variants was consistently confirmed at 10 fM in our studies, detailed investigation across a broad range of concentrations, extending up to the micromolar level, is required. It should be emphasized that the mature potato systemin sequence remains putative and has not been definitively confirmed via mass spectrometry, which represents a challenge in current plant peptidomics. Therefore, while the protective efficacy of these synthetic variants was demonstrated here, further incorporation of scrambled octadecapeptide systemin analogs and mock-inoculated controls will help clarify sequence-specific mechanisms versus non-specific effects. These future studies will help translate the observed reduction in PVY accumulation into practical agricultural applications utilizing the antiviral potential of synthetic systemin variants.

## 5. Conclusions

In summary, short synthetic elicitor peptides represent a developing area of peptide science and plant biology with potential applications in crop protection strategies. This study represents an initial evaluation of the antiviral capacity of synthetic variants of potato systemin—a peptide originally discovered as a stress signaling agent in response to wounding and herbivore attack. While this brief report highlights the necessity for further detailed investigations into the underlying molecular mechanisms, these preliminary findings suggest that short elicitor peptides could serve as potential components for developing eco-friendly antiviral strategies in agriculture.

## Data Availability

The original contributions presented in this study are included in the article. Further inquiries can be directed to the corresponding authors.

## Supplementary Materials

The following supporting information can be downloaded at: https://www.mdpi.com/article/10.3390/biom16091353/s1, Figure S1: Workflow for evaluating PVY^O^ accumulation via ELISA; Figure S2: Workflow for evaluating PVY^O^ accumulation via RT-qPCR; Figure S3: Workflow of the cumulative spraying regimen against the PVY^NTN^ strain; Figure S4: Relative expression levels of PVY^O^ RNA in potato plants.

## Abbreviations

ANOVA	Analysis of variance
antiSYS	Anti-systemin peptide
DPI	Days post-inoculation
ELISA	Enzyme-linked immunosorbent assay
Fmoc	9-Fluorenylmethoxycarbonyl
HSD	Honestly significant difference
HPLC	High-performance liquid chromatography
LRR	Leucine-rich repeat
MALDI-TOF/TOF-MS	Matrix-assisted laser desorption/ionization time-of-flight/time-of-flight mass spectrometry
nano-LC-MS/MS	Nano-liquid chromatography tandem mass spectrometry
PVY	Potato virus Y
PVY^O^	Potato virus Y, ordinary strain
PVY^NTN^	Potato virus Y, necrotic tuber ringspot strain
RT-qPCR	Real Time Quantitative Polymerase Chain Reaction
RP-HPLC	Reversed-phase high-performance liquid chromatography
SD	Standard deviation
SPPS	Solid-phase peptide synthesis
StSys I	*Solanum tuberosum systemin* I (octadecapeptide)
StSys I-ΔA	N-terminally truncated *Solanum tuberosum systemin* I (lacking the N-terminal Ala)
StSys I-ΔD	C-terminally truncated *Solanum tuberosum* systemin I (lacking the C-terminal Asp)
SYR1	Systemin receptor 1
SYR2	Systemin receptor 2
