# Supplementary material for: Synthetic Potato Systemin Enhances Solanum tuberosum Resistance to Potato Virus Y
Source: Biomolecules. 2026 Sep 17;16(9):1353. doi: 10.3390/biom16091353 (PMC13604349; doi:10.3390/biom16091353)
Supplement: Supplementary file 1 [file biomolecules-16-01353-s001.zip › biomolecules-4498326-supplementary.pdf]

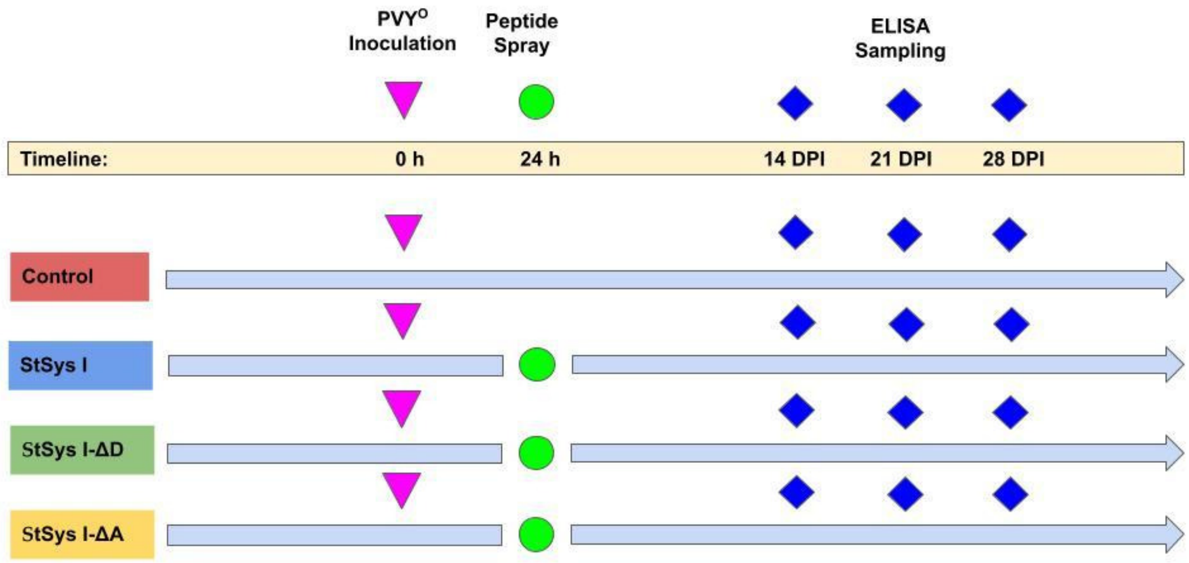

Fig. S1. Workflow for evaluating PVY^{O} accumulation via ELISA following post-infection treatment with synthetic StSys I and its truncated variants. Green circles (●) denote a single curative foliar application of StSys I variants administered 24 h post-inoculation. The purple triangle (▼) represents inoculation with the PVY^{O} strain (0 h). Blue diamonds (◆) indicate leaf sampling points performed at 14, 21, and 28 DPI for ELISA. The color-coded group labels correspond to the respective bar colors used in Fig. 1 of the main text.

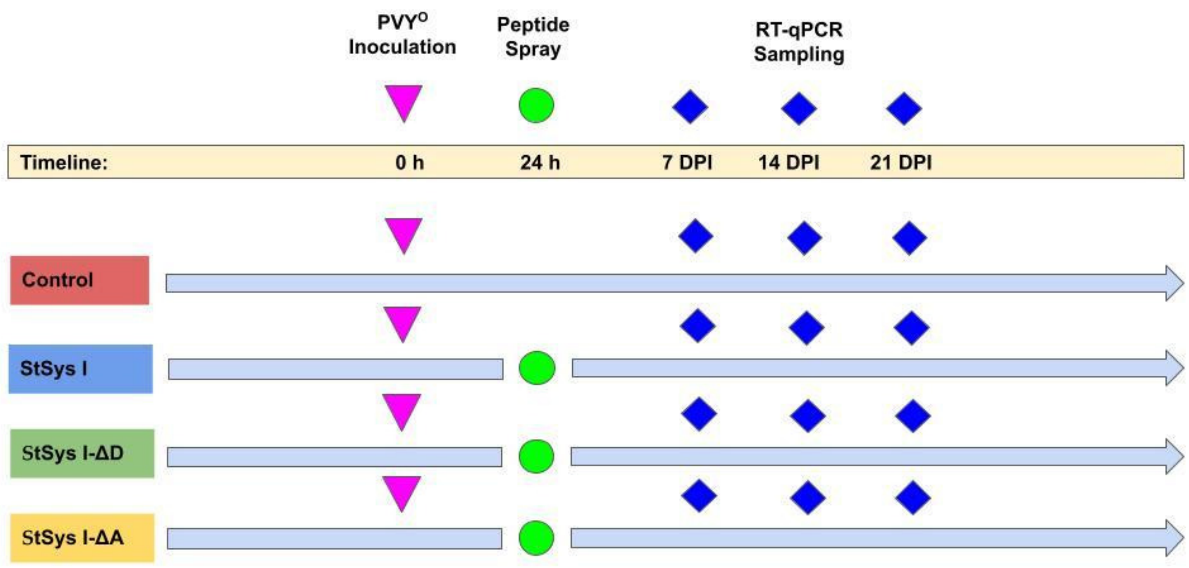

Figure S2. Workflow for evaluating PVY^{O} accumulation via RT-qPCR following post-infection treatment with synthetic StSys I and its truncated variants. Green circles (●) denote a single curative foliar application of StSys I variants administered 24 h post-inoculation. The purple triangle (▼) represents inoculation with the PVY^{O} strain (0 h). Blue diamonds (◆) indicate leaf sampling points performed at 7, 14, and 21 DPI for RT-qPCR analysis of viral RNA accumulation. The color-coded group labels correspond to the respective bar colors used in Fig. S4.

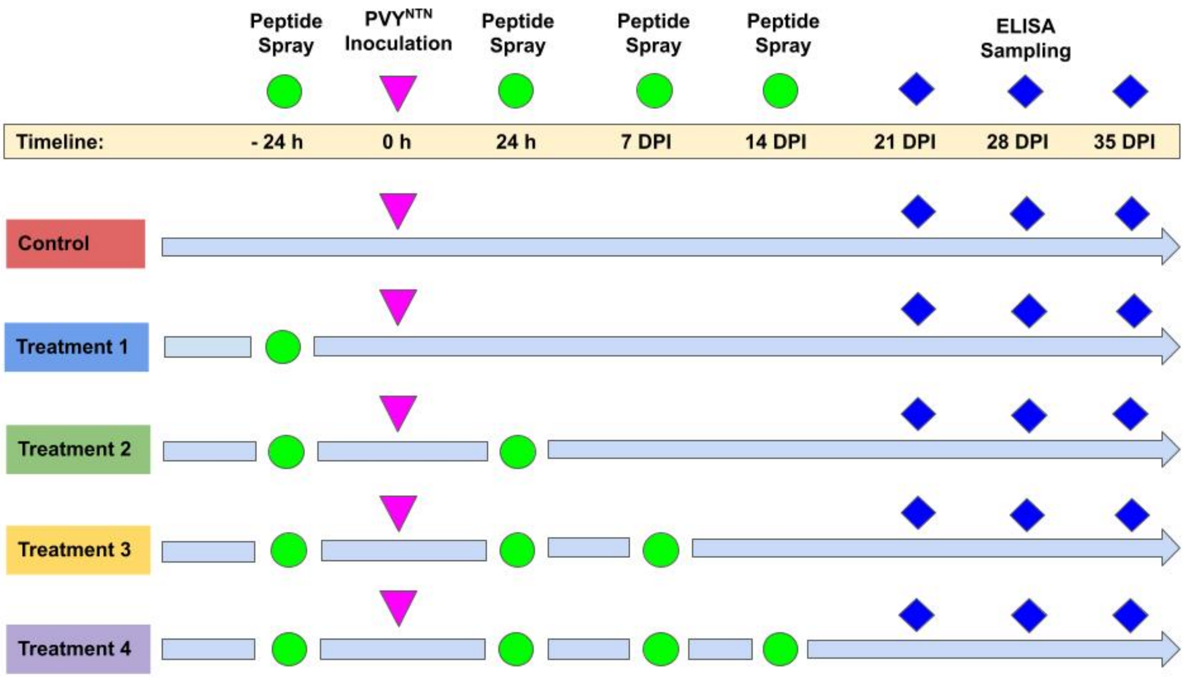

Fig. S3. Workflow of the cumulative spraying regimen against the PVY^{NTN} strain followed by ELISA. Green circles (●) denote the application of synthetic peptide StSys I, the purple triangle (▼) represents virus inoculation (0 h), and blue diamonds (◆) indicate leaf sampling points for ELISA at 21, 28, and 35 DPI. The color-coded group labels correspond to the respective bar colors used in Fig. 2 of the main text.

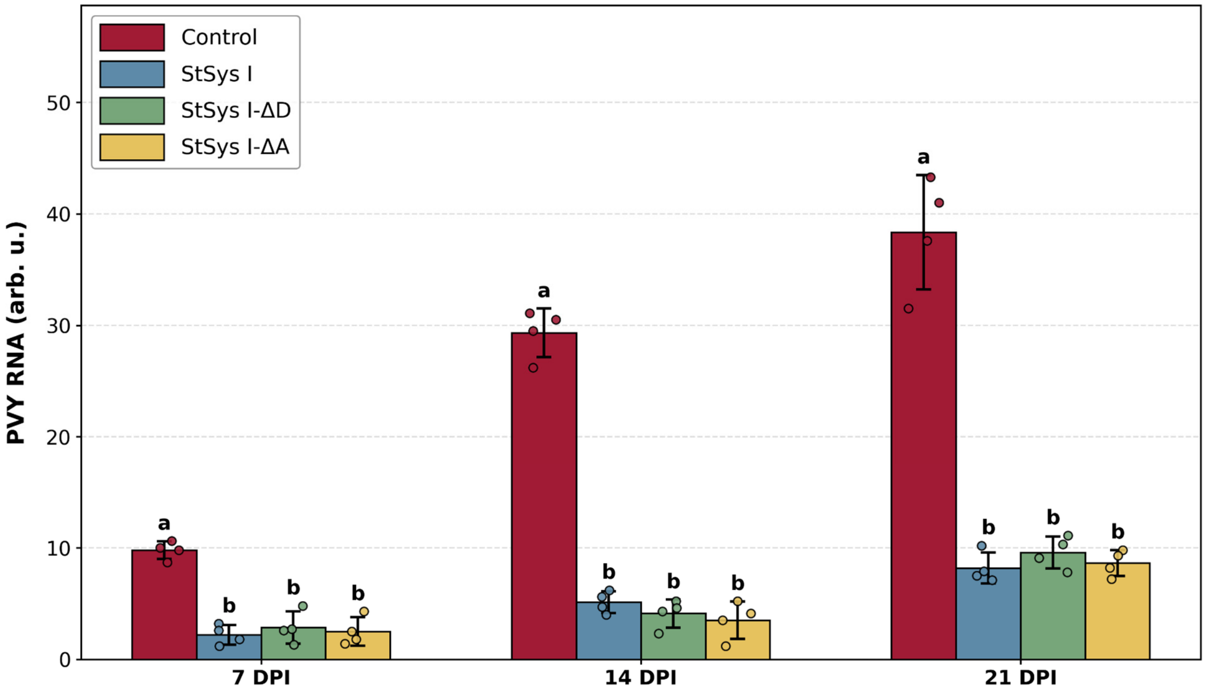

Fig. S4. Relative expression level of PVY^{O} RNA in potato plants (*Solanum tuberosum* cv. Indigo) at 7, 14, and 21 DPI quantified by RT-qPCR. Plants were treated curatively 24 h post-infection with the synthetic StSys I, its variants StSys I-ΔA and StSys I-ΔD (10 fM), or water at Control group (Fig S2) . Data represent mean ± SD from n = 4 independent biological replicates. Different letters indicate statistically significant differences ($p < 0.05$, Tukey HSD).
